# Synthesis and Guest Recognition of Switchable Pt-Salphen Based Molecular Tweezers

**DOI:** 10.3390/molecules23050990

**Published:** 2018-04-24

**Authors:** Lorien Benda, Benjamin Doistau, Bernold Hasenknopf, Guillaume Vives

**Affiliations:** Institut Parisien de Chimie Moléculaire, Sorbonne Université, CNRS UMR 8232, 4 Place Jussieu, 75005 Paris, France; lorien.benda@sorbonne-universite.fr (L.B.); benjamin.doistau@unige.ch (B.D.); bernold.hasenknopf@sorbonne-universite.fr (B.H.)

**Keywords:** molecular tweezers, guest binding, terpyridine, platinum, salphen

## Abstract

Molecular tweezers are artificial receptors that have an open cavity generated by two recognition units pre-organized by a spacer. Switchable molecular tweezers, using a stimuli-responsive spacer, are particularly appealing as prototypes of the molecular machines that combine mechanical motion and allosteric recognition properties. In this present study, the synthesis of switchable molecular tweezers composed of a central terpyridine unit substituted in 4,4″ positions by two Pt(II)-salphen complexes is reported. The terpyridine ligand can be reversibly converted upon Zn(II) coordination from a free ‘U’-shaped closed form to a coordinated ‘W’ open form. This new substitution pattern enables a reverse control of the mechanical motion compared to the previously reported 6,6″ substituted terpyridine-based tweezers. Guest binding studies with aromatic guests showed an intercalation of coronene in the cavity created by the Pt-salphen moieties in the closed conformation. The formation of 1:1 host-guest complex was investigated by a combination of NMR studies and DFT calculations.

## 1. Introduction

The concept of molecular tweezers was first introduced by Whitlock [1] who defined a molecular receptor characterized by two flat, generally aromatic, recognition sites pre-organized by a spacer to create an open cavity. Depending on the flexibility of the spacer, different recognition properties can be obtained. Rigid or semi-flexible spacers have so far been the most frequently used in the design of molecular tweezers, mostly for molecular recognition purposes [2,3,4,5,6,7,8,9,10]. However, stimuli-responsive spacers using redox [11,12], photochemical [13,14,15,16], pH [17,18,19,20] or ion coordination [21,22,23,24,25,26,27] stimuli have been recently used to control switchable molecular tweezers and create molecular machines or devices.

**Figure 1:**
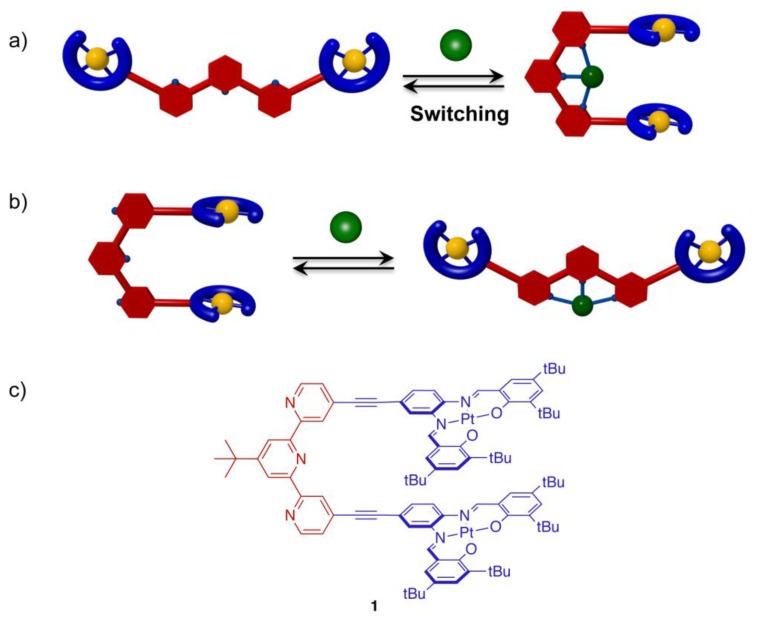
Schematic representation of the: (**a**) previous and (**b**) new switchable molecular tweezers; (**c**) molecular structure of synthetized tweezers.

We have developed a family of switchable molecular tweezers based on a terpyridine unit substituted by metal salphen complexes with different properties depending on the metallic ion coordinated to the salphen ligand. By using a 6,6″ substituted terpyridine, the tweezers can switch upon metal coordination from a “W”-shaped open form to a “U” closed form, which brings the two salphen moieties in close spatial proximity (Figure 1). This controlled and large modification of the distance between the two functional units has been successfully applied to modulate magnetic [28,29] or redox properties [30] using Cu(II) and Ni(II) salphen complexes, respectively. Square planar Pt(II) luminophores present attractive properties, such as tunable excited states that are highly sensitive to their microenvironment [31]. In particular, Pt(II)-salphen derivatives have been reported to exhibit high quantum efficiencies under ambient conditions [32,33,34,35,36,37]. Thus, luminescent switchable tweezers based on Pt(II)-salphen were developed to act as an efficient probe for guest intercalation. Although selective binding of Hg(II) coupled with a dramatic luminescence quenching was obtained in the closed form, no intercalation of flat aromatic substrates was observed [38,39]. This low affinity was attributed to the steric hindrance in the binding site caused by the cation coordinated to the terpyridine unit, which inhibits the binding of guests. To prevent the intercalation of the metal ion in the closed conformation, a new 4,4″-disubstituted terpyridine ligand was designed (Figure 1c). This new substitution pattern should result in a default ‘U’-shaped closed conformation, which can be converted upon metal coordination to an open ‘W’-shaped form. The closed conformation should enhance guest binding as observed with similar molecular clips bearing Pt-terpyridine complexes [8,40,41,42]. Herein we present the synthesis of Pt-based molecular tweezers as well as their switching mechanism and guest binding properties.

## 2. Results and Discussion

### 2.1. Synthesis

**Scheme 1:**
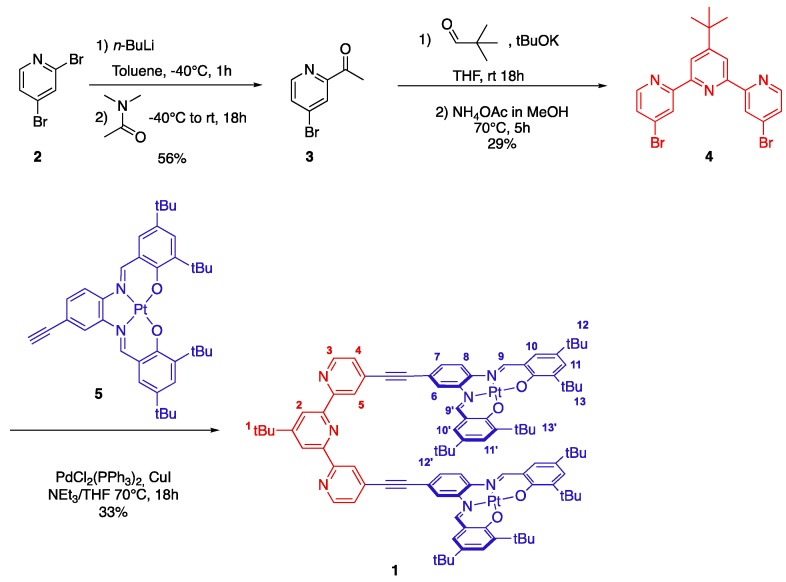
Synthesis of molecular tweezers **1**.

The synthesis of tweezers **1** is based on a modular approach, which uses a double Sonogashira coupling reaction between 4,4″ substituted di-bromo-terpyridine **4** and alkyne substituted Pt(II)-salphen complex **5** as a key step (Scheme 1). Since Pt(II)-salphen complexes are inert, they can be used as building blocks in cross-coupling reactions, which enables the control of the coordination sphere of the two different salphen and terpyridine ligands. The synthesis of the 4,4″ substituted terpyridine started from 2,4-dibromopyridine **2,** which was acetylated by a regioselective lithium halogen exchange reaction with *n*-butyllithium followed by a reaction with *N*,*N*-dimethylacetamide [43]. After hydrolysis and purification by column chromatography, **3** was obtained in 56% yield. The terpyridine ligand **4** was then synthetized using a one-pot Hantzsch-type procedure [44]. Actetyl-pyridine **3** was reacted with pivalaldehyde in the presence of potassium *tert*-butoxide as base in THF to form an intermediate diketo product, which was subsequently cyclized by ammonium acetate and oxidized by air. After purification by column chromatography on neutral aluminum oxide, 4,4″-dibromoterpyridine **4** was obtained in 29% yield.

In the last step, the terpyridine unit **4** was subjected to a double Sonogashira coupling reaction with the alkyne substituted Pt-salphen complex **5** [38]. Tweezers **1** were obtained in 33% yield and were fully characterized by NMR spectroscopy and mass spectrometry. No exchange between Pd(II) or Cu(I) and Pt(II) was detected, confirming the non-lability of Pt(II)-salphen complexes and validating our ‘chemistry on complex’ strategy.

### 2.2. Switching Studies

**Figure 2:**
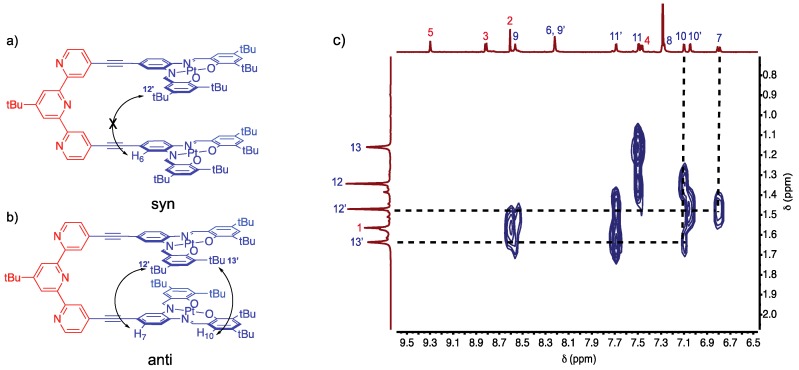
(**a**,**b**) Molecular structure of the *syn*- and *anti*-conformations and (**c**) ^1^H NOESY Spectrum (400 MHz, 300 K) of **1** in CDCl_3_ showing the correlation peaks between H-7/H-12′ and H-10/H-13′ indicative of a closed stacked *anti*-conformation.

**Figure 3:**
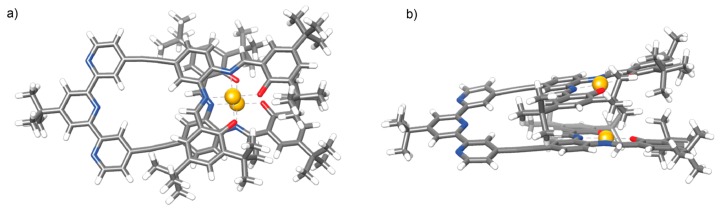
DFT-optimized structure of **1** (B3LYP/6-31G**/LanL2DZ); (**a**) top view and (**b**) side view.

The conformation of tweezers **1** was first investigated by ^1^H NMR spectroscopy. The 2D NOESY spectrum in CDCl_3_ is consistent with a ‘U’-shaped geometry of the terpyridine ligand with the absence of correlation between the H-2 and H-5 protons (see Figure S1). This is expected due to the electronic repulsion between the nitrogen lone pairs favoring the s-*trans* conformation. Due to the dissymmetric substitution pattern of the Pt-salphen moiety, the closed conformation can exist as *syn*- or *anti*-conformers (Figure 2a,b). The cross-peaks between H-7 and H-12′ and between H-10 and H-13′ (Figure 2c) indicate a short distance between the two Pt-salphen complexes, which can be observed only if the two moieties adopt the *anti*-conformation. To obtain a better insight into the conformation of the tweezers, DFT calculations were performed. In the U-shaped form, the terpyridine ligand adopts a slightly twisted geometry, resulting in a folded helical structure with the two arms in the *anti*-conformation crossing at the level of the salphen units (Figure 3). The resulting minimal distance between H-7 and H-12′ is approximately 2.3 Å, which explains the obtained correlation in the NOESY spectrum. DFT calculations were also performed for the open W-shaped form. In the optimized structure, the terpyridine ligand is not co-planar as each pyridine moiety displays an average dihedral angle of 32° to minimize the repulsion between the nitrogen lone pairs (Figure S2). As expected, the energy of the W form is higher than the U-shaped one of around 80 kJ·mol^−1^. This significant difference is probably due to a combination of the destabilization of the *s-cis* conformation of the terpyridine unit in the W-shaped form and a stabilizing interaction between the two Pt-salphen units in the closed U-shaped conformation.

**Figure 4:**
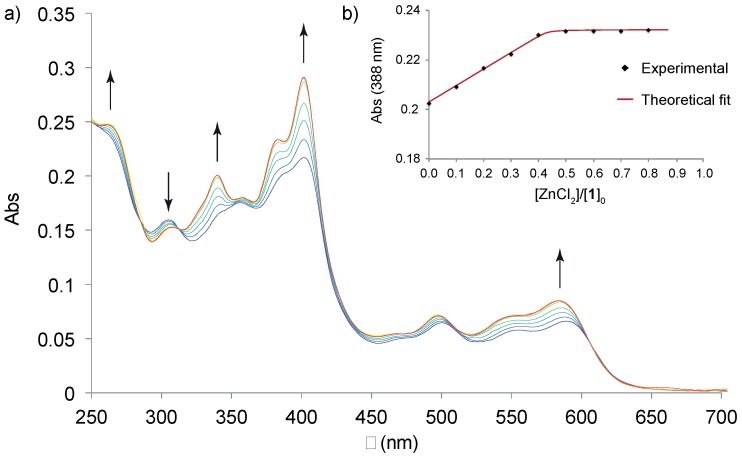
(**a**) UV-Vis titration of **1** (5.0 × 10^−6^ mol·L^−1^) by ZnCl_2_ in CHCl_3_ and (**b**) Absorption at 388 nm and fitting with a 2:1 binding model.

**Figure 5:**
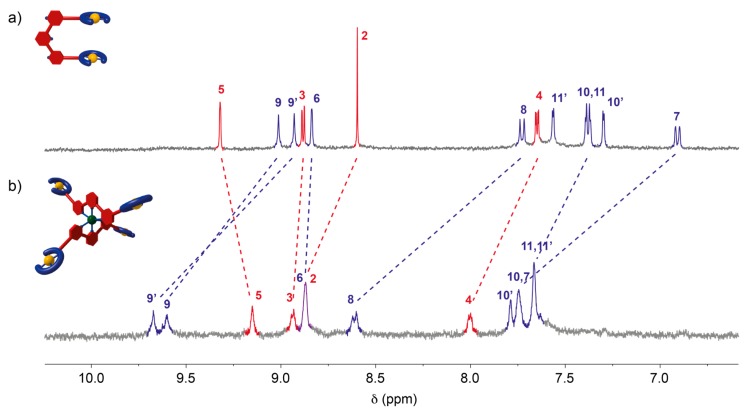
^1^H NMR spectra (400 MHz, 300 K) in DMSO-d_6_ of: (**a**) tweezers **1** and (**b**) [Zn(**1**)_2_]Cl_2_.

The opening of tweezers **1** was monitored by UV-Vis spectroscopy in chloroform. Titration of **1** with ZnCl_2_ (Figure 4) showed a single evolution up to 0.5 equivalents of Zn^2+^ with isosbestic points at the three curve crossings (λ = 605, 310 and 286 nm). This is consistent with an equilibrium between only two species corresponding to free and coordinated tweezers. The linear evolution with a sharp endpoint at 0.5 eq. of Zn^2+^ indicates the formation of the bis-terpyridine 2:1 complex [Zn(**1**)_2_]^2+^. The formation of a 2:1 complex was confirmed by mass spectrometry with a signal at 1834 *m*/*z*, which corresponds to di-cationic [Zn(**1**)_2_]^2+^ species (Figure S5). Fitting of the titration curve with a 2:1 model revealed very strong association constants (log K_1_, K_2_ > 8), which are similar to those previously observed with terpyridine ligands [45,46]. The switching was also monitored by ^1^H-NMR in DMSO-d_6_ due to the low solubility of [Zn(**1**)_2_]Cl_2_ in CDCl_3_, toluene or CDCl_3_/CD_3_CN mixtures. Upon addition of ZnCl_2_, progressive disappearance of the signals of closed tweezers **1** is observed with only one set of new signals appearing corresponding to [Zn(**1**)_2_]Cl_2_ (Figure S3). An excess of ZnCl_2_ was necessary to fully convert **1** to [Zn(**1**)_2_]Cl_2_ due to the competition of the solvent coordination with Zn^2+^ [47,48]. The spectrum of open tweezers (Figure 5b) is characteristic of a coordinated terpyridine ligand with downfield shifts for meta-protons H-2 and H-4 compared to the open tweezers. Large shifts are also observed for the protons of the Pt-salphen moieties. In particular, H-7 and H-8 protons in addition to H-9 and H-9′ imine protons are strongly deshielded. The open conformation prevents the intramolecular stacking between the Pt-salphen units and results in a loss of magnetic anisotropy effects present in the closed conformation. The 2D NOESY spectrum is consistent with a W-shaped geometry of the terpyridine ligand with a correlation peak between H-2 and H-5 protons (Figure S4).

**Figure 6:**
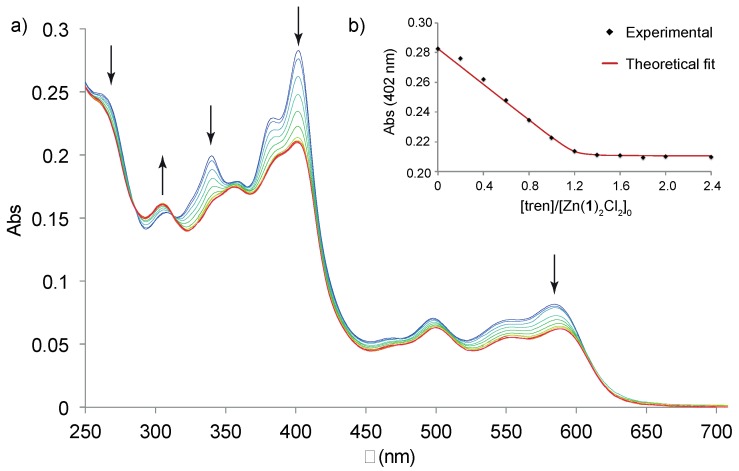
(**a**) UV-Vis titration of [Zn(**1**)_2_]Cl_2_ (5.0 × 10^−6^ mol·L^−1^) upon the addition of tren in CHCl_3_, after the opening with 0.5 equivalent of ZnCl_2_ and (**b**) Absorption at 402 nm and fitting with a 1:1 binding model.

The reversibility of the molecular motion was investigated by addition of a competitive ligand to remove the Zn^2+^ from the terpyridine. Tris 2-aminoethyl amine (tren) was chosen due to its high binding constant with metallic cations [49]. The closing of the tweezers previously opened with Zn^2+^ was investigated by UV-Vis titration (Figure 6). A complete reclosing was observed after the addition of around 1.2 eq of tren. The presence of isosbestic points at all curve crossings (λ = 310 and 286 nm) is consistent with only two absorbing species being in equilibrium (open and closed) with the reclosing following the reverse path of the opening. This demonstrates the reversible working operation of tweezers **1** that can be opened and closed with the opposite stimuli to those used for the 6,6″ substituted terpyridine based tweezers that we have previously reported [38,39].

### 2.3. Guest-Binding Studies

**Figure 7:**
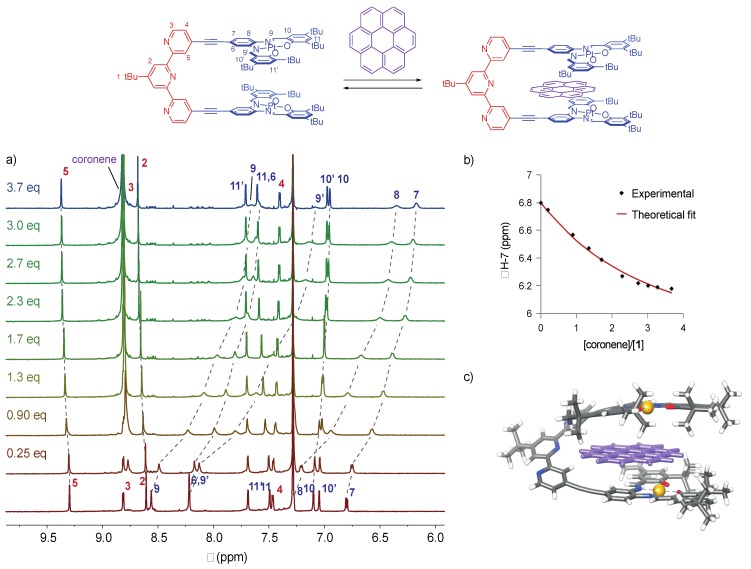
(**a**) ^1^H-NMR (600 MHz) titration of Tweezers **1** (2.0 mM) with coronene in CDCl_3_ at 300 K; (**b**) Fitting with a 1:1 binding isotherm model; and (**c**) DFT-optimized structure of the host guest complex [coronene ⊂ **1**] (B3LYP/6-31G**/LanL2DZ).

The recognition and intercalation abilities of tweezers **1** towards flat aromatic guest molecules were then investigated by analogy with bis(Pt-terpyridine) molecular clips, which have been reported in the literature [8,9,40,42]. Titration experiments were monitored by ^1^H NMR in CDCl_3_ at 300 K. Upon addition of coronene, large upfield shifts for phenylene H-7, H-8 and H-6 protons (Δδ of ca. −0.6 to −0.9 ppm) as well as H-9 and H-9′ imine protons (Δδ of ca. −1.1 ppm) were observed (Figure 7a), which are probably due to the π-stacking interactions between the salphen moiety and the coronene. In addition, the signal of coronene was also shifted upfield by approximately 0.2 ppm. The protons of the terpyridine unit, H-3 and H-4, are less affected. Only the H-5 and H-2 protons showed a small downfield shift (Δδ of ca. +0.1 ppm), indicating weak interactions between the coronene and the terpyridine unit. These shifts are consistent with recognition taking place inside the cavity formed by the two Pt-salphen units. Upon lowering the temperature in CD_2_Cl_2_, the broadening of the NMR signals was observed for a 1:1.5 mixture of **1** and coronene, with a coalescence that was reached at around 220 K (Figure S6). At 190 K, two sets of signals were observed for all peaks, which indicates the transition from a fast to slow exchange upon cooling. Since the major set of signals have the same chemical shifts as the free tweezers at 190 K, the minor ones can be attributed to the host/guest complex.

To obtain a better insight into the interaction between coronene and **1**, 2D NOESY experiments on a 1:1.5 mixture of tweezers **1** and coronene were performed. No correlation between the coronene protons and **1** were observed in NOESY, which is probably due to the fast exchange and relatively low binding constant. However, the extinction of the correlations between H-7 and H-12′ and between H-10 and H-13′ (Figure S7) is indicative of an increased distance between the two Pt-salphen units as expected from the intercalation of coronene. The formation of a 1:1 complex [coronene ⊂ **1**] was confirmed by mass spectrometry, with characteristic peaks at 2101.9, 2123.8 and 2139.8 m/z (see Figure S8) corresponding to the mono-charged species [coronene ⊂ **1** + H]^+^, [coronene ⊂ **1** + Na]^+^ and [coronene ⊂ **1** + K]^+^, respectively. The binding constant between the coronene and tweezers **1** was determined by fitting the NMR titration data with a 1:1 binding model [50] (Figure 7b). The obtained value (K = 300 ± 15 M^−1^) is moderate compared to the aromatic binding reported in the literature with bis(Pt-terpyridine) molecular clips [6,40,42]. This is probably due to the bulky tert-butyl groups introduced for solubility reasons, which prevent a strong π-stacking interaction between the Pt-salphen moieties and the coronene. However, it should be noted that the bis(Pt-terpyridine) molecular clips reported by Yam or Bosnich [6,40,42] use cationic Pt-terpyridine complexes as recognition units. Compared to the neutral Pt-salphen units of tweezers **1**, the electron poor and charged units might improve the binding with aromatic substrates via enhanced donor/acceptor and ion/π interactions.

Diffusion ordered NMR spectroscopy (DOSY) experiments were also performed in CDCl_3_ at 300 K to provide additional evidence for the binding of coronene. The diffusion coefficients of tweezers **1** and coronene are 9.06 × 10^−10^ m^2^·s^−1^ and 1.38 × 10^−9^ m^2^·s^−1^, respectively, whereas the 1:1.5 mixture presents values of 6.79 × 10^−10^ m^2^·s^−1^ for **1** and 1.31 × 10^−9^ m^2^·s^−1^ for the coronene. The diffusion coefficient of the coronene is slightly lowered in the mixture, which indicates that a non-negligible amount of the guest is accommodated in the tweezers. The diffusion constant of Tweezers **1** is also significantly reduced by the presence of coronene, which suggests an increase in the hydrodynamic radius as expected from the intercalation of the guest that moves the Pt-salphen moieties apart. The exact binding mode for the complex [coronene ⊂ **1**] was further clarified by DFT calculations (Figure 7c). In the optimized structure, the coronene is intercalated between the two Pt-salphen units with inter-planar π-distances that were calculated to be approximately 3.8 Å. The close proximity between the coronene and the diamino-phenylene and imine moieties of the salphen is consistent with the remarkable upfield shifts of NMR signals for the corresponding protons. The distance between the two salphens increased from 4.5 to 7.6 Å in the presence of the guest, which is in agreement with the increased hydrodynamic radius observed by DOSY as well as with the absence of correlation between protons H-7 and H-12′ and between H-10 and H-13′ in NOESY.

The release of the coronene guest by opening the tweezers with Zn(II) was also investigated. Whereas the coronene recovers the chemical shift of the free species upon the addition of ZnCl_2_ to a solution of [coronene ⊂ **1**] in CDCl_3_, the formed zinc complex becomes insoluble and precipitates. This indicates that a release of the guest occurs as expected from the opening, but the concomitant precipitation of the open tweezers precludes its unambiguous attribution to the mechanical motion. Different Zn(II) salts or solvent mixtures were tried but unfortunately, no combination was found where all species (coronene and zinc tweezers) remained soluble at the NMR concentration.

Finally, the binding of smaller aromatic guests was examined. Upon addition of perylene, small shifts were observed by NMR (Figure S9), indicating some intercalation. However, the binding constant was too low to be accurately determined. Surprisingly, the addition of a Pt-salphen complex where Pt-Pt interactions should provide an additional driving force [41,51] did not result in any intercalation. The *tert*-butyl groups on the Pt-salphen units introduced for solubility reasons are probably preventing optimal positioning of the guest to combine π-stacking and Pt-Pt interactions.

## 3. Materials and Methods

### 3.1. General Procedures

Reagent grade tetrahydrofuran was distilled from sodium and benzophenone. Tetrahydrofuran and triethylamine were degassed by three freeze–pump–thaw cycles before being used in the Sonogashira coupling reactions. All other chemicals were purchased from commercial suppliers and used without further purification. Complex **5** was synthetized according to the literature [38]. Flash column chromatography was performed using silica gel from Merck (40–63 µm) or GraceResolv High Resolution Flash Cartridges (particle size of 40 µm). Thin layer chromatography was performed using aluminum plates pre-coated with silica gel or neutral aluminum oxide 60 F254 purchased from VWR, which had a 0.20-mm layer thickness. Absorption spectra were recorded on a JASCO V-670 spectrophotometer. Infrared spectra were recorded on a Bruker tensor 27 ATR spectrometer. Electrospray ionization (ESI) mass spectrometry was performed on a Bruker microTOF spectrometer.

### 3.2. Synthesis


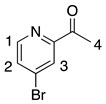


*4-Bromo-2-acetylpyridine* **3** [43]. In a round bottom flask, 2,4-dibromopyridine **2** (4.0 g, 17 mmol, 1 eq) was dissolved in 160 mL of dry toluene. The solution was cooled down to −40 °C and *n*-Buli (11 mL of a solution at 1.6 M in hexane, 17 mmol, 1 eq) was added dropwise. The mixture was stirred for 1.5 h at −40 °C. *N*,*N*-dimethylacetamide (2.6 g, 30 mmol, 1.8 eq) was added and the mixture was allowed to return to room temperature and stirred for 1 h. A saturated solution of NH_4_Cl (around 50 mL) was added and the organic phase was separated. The aqueous phase was extracted with CHCl_3_ and the combined organic phases were dried over MgSO_4_. The solvents were evaporated under reduced pressure, before the crude product was purified by column chromatography (SiO_2_: from Cyclohexane/EtOAc (70/30) to EtOAc (100)), yielding **3** as a white solid (1.83 g, 56%). ^1^H NMR (400 MHz, 300 K, CDCl_3_) *δ* 8.51 (dd, *J* = 5.2, 0.6 Hz, 2 H, H_1_), 8.20 (dd, *J* = 1.8, 0.6 Hz, 1 H, H_3_) 7.65 (dd, *J* = 1.8, 5.2 Hz, 1 H, H_2_), 1.59 (s, 3 H, H_4_); ^13^C NMR (100 MHz, 300 K, CDCl_3_) *δ* 198.70, 154.30, 149.69, 134.11, 130.19, 125.27, 25.83.


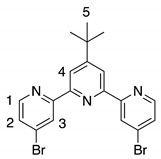


4*,4″-Dibromo-4′-(tert-butyl)-2,2′:6′,2″-terpyridine* **4**. 4-bromo-2-acetylpyridine **3** (700 mg, 3.50 mmol, 2 eq) was added to a suspension of *t*-BuOK (590 mg, 5.25 mmol, 3 eq) in THF (25 mL). Pivalaldehyde (150 mg, 1.75 mmol, 1 eq) was added and the mixture was stirred at room temperature for 18 h. A solution of NH_4_OAc (1.50 g, 19.25 mmol, 11 eq) in MeOH (10 mL) was introduced, before the mixture was heated at 70 °C for 5 h. After solvent evaporation, the crude product was purified by a short column chromatography (Al_2_O_3_: Cyclohexane/Ethyl Acetate (96/4)), yielding dibromo-terpyridine **4** as a white solid (256 mg, 29%). ^1^H NMR (400 MHz, 300 K, CDCl_3_) *δ* 8.76 (dd, *J* = 0.4, 2.0 Hz, 2 H, H_3_), 8.53 (dd, *J* = 0.4, 5.3 Hz, 2 H, H_1_), 8.51 (s, 2 H, H_4_), 7.51 (*J* = 2.0, 5.3 Hz, 2 H, H_2_), 1.46 (s, 9 H, H_5_); ^13^C NMR (100 MHz, 300 K, CDCl_3_) *δ* 162.68, 157.83, 154.38, 149.93, 134.04, 127.02, 124.83, 119.26, 35.58, 30.85. ESI-HRMS *m/z*: [M+Na]^+^ calculated (C_19_H_17_N_3_Br_2_Na): 469.9662, found: 469.9667.


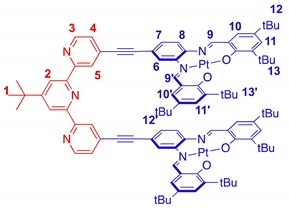


*Tweezers* **1**. In a Schlenk tube, terpyridine **4** (39 mg, 0.087 mmol, 1 eq), complex **5** (263 mg, 0.35 mmol, 4 eq), PdCl_2_(PPh_3_)_2_ (12 mg, 0.017 mmol, 20 mol%) and CuI (7 mg, 0.035 mmol, 40 mol%) were introduced and placed under an Argon atmosphere. After this, a mixture of NEt_3_ (5 mL)/THF (10 mL) that was previously distilled and degassed by freeze pump thaw cycles was added. The mixture was stirred at 70 °C under argon for 18 h. After solvent evaporation, the purple crude product was finally purified by column chromatography (SiO_2_: from cyclohexane/dichloromethane (50/50) to dichloromethane/methanol (92/8)), yielding tweezers **1** as a purple solid (52 mg, 33%). ^1^H NMR (400 MHz, CD_2_Cl_2_) *δ* of 9.33 (dd, *J* = 0.7, 1.5 Hz, 2 H, H_5_), 8.79 (dd, *J* = 0.7, 5.0 Hz, 2 H, H_3_), 8.61 (s, 2 H, H_2_), 8.57 (s, 2 H, H_9_), 8.27 (d, *J* = 1.2 Hz, 2 H, H_6_), 8.25 (s, 2 H, H_9′_), 7.70 (d, *J* = 2.2 Hz, 2 H, H_11′_), 7.48 (m, 4 H, H_11-4_), 7.28 (d, *J* = 8.6 Hz, 2 H, H_8_), 7.12 (d, *J* = 2.5 Hz, 2 H, H_10_), 7.05 (d, *J* = 2.2 Hz, 2 H, H_10′_), 6.82 (dd, *J* = 1.2, 8.6 Hz, 2 H, H_7_), 1.61 (s, 18 H, H_13′_), 1.53 (s, 9 H, H_1_), 1.46 (s, 18 H, H_12′_), 1.33 (s, 18 H, H_12_), 1.13 (s, 18 H, H_13_); ^13^C NMR (100 MHz, CD_2_Cl_2_) δ 165.29, 164.53, 163.66, 157.10, 154.80, 150.21, 149.64, 147.74, 146.36, 145.30, 141.83, 141.70, 137.55, 137.44, 132.51, 131.66, 131.51, 129.59, 129.47, 128.91, 125.76, 124.40, 121.37, 120.99, 119.72, 118.42, 114.83, 94.30, 89.83, 36.81, 36.34, 36.06, 34.58, 34.35, 31.68, 31.53, 31.14, 30.62, 29.95. ESI-HRMS *m*/*z*: [M + Na]^+^ calculated (C_95_H_107_N_7_Pt_2_O_4_): 1823.759, found: 1823.7589.

### 3.3. Titration Procedures

^1^H NMR titrations were performed using CDCl_3_ dried over molecular sieves (4 Å), which were passed through dried neutral aluminum oxide. Metal salts and tris(2-aminoethyl)amine were used without any purification. All solutions of tweezers, metal salts and ligands used for titrations were prepared in volumetric flasks, while the additions were made with Hamilton syringes.

Tweezers **1** opening: To 0.5 mL of closed tweezers **1** (1.0 × 10^−3^ M) dissolved in DMSO-*d*_6_ in an NMR tube (5 mm), were added 0.2 eq of ZnCl_2_ (4 μL of a 2.5 × 10^−2^ M solution in D_3_CCN). After each addition, the tube was heated at reflux during 5 s, then cooled at room temperature, and the ^1^H NMR spectrum was recorded.

Guest binding: To 0.5 mL of closed tweezers **1** (2.0 × 10^−3^ M) dissolved in CDCl_3_ in an NMR tube (5 mm), were added coronene as a solid. After each addition, the tube was heated at reflux during 5 s, then cooled at room temperature, and the ^1^H NMR spectrum was recorded.

UV-visible absorption spectra were recorded on a JASCO V-670 spectrophotometer at 25 °C. CHCl_3_ was dried over molecular sieves with a size of 4 Å and neutralized on neutral Al_2_O_3_. Metal salts were used without any purification. The solutions of tweezers and metals salts used for titrations were prepared in volumetric flasks, before the additions were made with Hamilton syringes. The metal salt concentrations in the stock solutions were checked by titration with a terpyridine solution. Curve fitting were performed by a non-linear least-squares fit of the absorbance compared to the concentration of guest added using the Matlab program developed by Thordarson [50].

The titrations monitored by UV-Visible spectroscopy have been performed according to the following general procedure:

Tweezers **1** opening: To 3.0 mL of open tweezers (5.0 × 10^−6^ M) dissolved in CHCl_3_ in a quartz cell (10 mm), were added 0.1 eq ZnCl_2_ (3 μL of 1.0 × 10^−3^ M solution in H_3_CCN). After each addition, a UV-Visible absorption spectrum (250–700 nm, 400 nm/min, 25 °C) was recorded.

[Zn(**1**)_2_]Cl_2_ closing: To 3.0 mL of closed tweezers (5.0 × 10^−6^ M) dissolved in CHCl_3_ in a quartz cell (10 mm), were added 0.1 eq tren (3 μL of 1.0 × 10^−3^ M solution in H_3_CCN). After each addition, a UV-Visible absorption spectrum (250–700 nm, 400 nm/min, 25 °C) was recorded.

### 3.4. Computational Details

Calculations were performed with the Gaussian 09 software [52]. Complete geometry optimizations were carried out using the density functional theory method with the conventional Becke-3-Lee-Yang-Parr (B3LYP) exchange-correlation functional and 6-31G**/LanL2DZ. The platinum atoms were modeled using the effective core potential and the corresponding valence orbitals LanL2DZ in order to decrease the number of basis functions. The other atoms were described by the double zeta 6-31G** base, which takes into account the polarization orbitals of all atoms, including hydrogen atoms. Vibrational analysis was performed at the same level in order to check the obtaining of a minimum on the potential energy surface.

## 4. Conclusions

In conclusion, coordination-based switchable molecular tweezers with Pt-salphen moieties have been synthetized. By using an original 4,4″ substituted terpyridine unit, the tweezers adopt a free ‘U’-shaped closed conformation that can be reversibly converted by metal coordination to an open ‘W’-shaped one. This new substitution pattern gives access to an opposite control of the mechanical motion compared to the previously reported 6,6″ substituted switchable tweezers. The cavity formed by the Pt-salphen in the closed conformation enabled the binding of a coronene guest with the formation of a 1:1 complex. Such intercalation of an aromatic substrate was not possible in previous tweezers due to the presence of a cation near the cavity in the closed form. This demonstrates the interest of this new design, which will be exploited to achieve M-M interactions with guest complexes after further optimization of the binding sites.

## Supplementary Materials

Supplementary materials are available online http://www.mdpi.com/1420-3049/23/5/990/s1.
