# Supplementary material for: Synthesis and Guest Recognition of Switchable Pt-Salphen Based Molecular Tweezers
Source: Molecules. 2018 Apr 24;23(5):990. doi: 10.3390/molecules23050990 (PMC6102593; doi:10.3390/molecules23050990)
Supplement: Supplementary file 1 [file molecules-23-00990-s001.pdf]

Supporting Information

Synthesis and Guest Recognition of Switchable Pt-salphen based Molecular Tweezers

Lorien Benda [1], Benjamin Doistau [1], Bernold Hasenknopf [1] and Guillaume Vives [1,*]

[1] Sorbonne Université, CNRS UMR 8232, Institut Parisien de Chimie Moléculaire,

4 place Jussieu, 75005, Paris, France;

General procedures 2

Synthesis 2

Titration procedures 4

Computational details 4

Switching studies 5

Guest-binding studies 8

References 10

NMR spectra of compounds 11

General procedures

Reagent grade tetrahydrofuran was distilled from sodium and benzophenone. Tetrahydrofuran and triethylamine were degassed by three freeze-pump-thaw cycles before being used in the Sonogashira coupling reactions. All others chemicals were purchased from commercial suppliers and used without further purification. Complex **5** was synthetized according to the literature.[1] Flash column chromatography was performed using silica gel from Merck (40-63 µm) or GraceResolv High Resolution Flash Cartridges (particle size 40 µm). Thin layer chromatography was performed using aluminium plates pre-coated with silica gel or neutral aluminum oxide 60 F254 0.20 mm layer thickness purchased from VWR. Absorption spectra were recorded on a JASCO V-670 spectrophotometer. Infrared spectra were recorded on a Bruker tensor 27 ATR spectrometer. Electrospray ionisation (ESI) mass spectrometry was performed on a Bruker microTOF spectrometer.

Synthesis

4-bromo-2-acetylpyridine **3**[2]

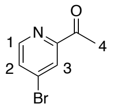

In a round bottom flask 2,4-dibromopyridine **2** (4.0 g, 17 mmol, 1 eq) was dissolved in 160 mL of dry toluene. The solution was cooled down to -40°C and *n*-Buli (11 mL of a solution at 1.6 M in hexane, 17 mmol, 1 eq) was added dropwise. The mixture was stirred during 1.5 h at -40°C. N,N-dimethylacetamide (2.6 g, 30 mmol, 1.8 eq) was added and the mixture was allowed to return to room temperature and stirred for 1 h. A saturated solution of NH_4Cl (around 50 mL) was added and the organic phase was separated. The aqueous phase was extracted with $CHCl_3$ and the combined organic phases were dried over $MgSO_4$. The solvents were evaporated under reduced pressure, and the crude product was purified by column chromatography (SiO_2: from Cyclohexane/EtOAc (70/30) to EtOAc (100)) yielding **3** as a white solid (1.83 g, 56%).

^{1}H NMR (400 MHz, 300 K, $CDCl_3$) δ 8.51 (dd, J = 5.2, 0.6 Hz, 2H, H_1), 8.20 (dd, J=1.8, 0.6 Hz, 1H, H_3), 7.65 (dd, J= 1.8, 5.2 Hz, 1H, H_2), 1.59 (s, 3H, H_4)

^{13}C NMR (100 MHz, 300 K, $CDCl_3$) δ 198.70, 154.30, 149.69, 134.11, 130.19, 125.27, 25.83

4,4''-Dibromo-4'-(tert-butyl)-2,2':6',2''-terpyridine **4**[3]

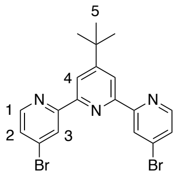

4-bromo-2-acetylpyridine **3** (700 mg, 3.50 mmol, 2 eq) was added to a suspension of *t*-BuOK (590 mg, 5.25 mmol, 3 eq) in THF (25 mL). Pivalaldehyde (150 mg, 1.75 mmol, 1 eq) was added and the mixture was stirred at rt during 18 h. A solution of NH_4OAc (1.50 g, 19.25 mmol, 11 eq) in MeOH (10 mL) was introduced, and the mixture was heated at 70 °C during

5 h. After solvent evaporation, the crude product was purified by a short column chromatography (Al_2O_3: Cyclohexane/Ethyl Acetate (96/4)) yielding dibromo-terpyridine **4** as a white solid (256 mg, 29%).

^{1}H NMR (400 MHz, 300 K, $CDCl_3$) δ 8.76 (dd, J = 0.4, 2.0 Hz, 2H, H_3), 8.53 (dd, J = 0.4, 5.3 Hz, 2H, H_1), 8.51 (s, 2H, H_4), 7.51 (J = 2.0, 5.3 Hz, 2H, H_2), 1.46 (s, 9H, H_5)

^{13}C NMR (100 MHz, 300 K, $CDCl_3$) δ 162.68, 157.83, 154.38, 149.93, 134.04, 127.02, 124.83, 119.26, 35.58, 30.85

ESI-HRMS *m/z* (%): $[M+Na]^+$ calc ($C_{19}H_{17}N_3Br_2Na$): 469.9662, found: 469.9667

*Tweezers **1***

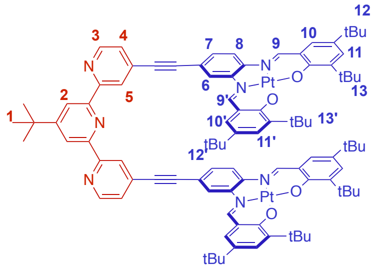

In a Schlenk tube, terpyridine **4** (39 mg, 0.087 mmol, 1 eq), complex **5** (263 mg, 0.35 mmol, 4 eq), $PdCl_2(PPh_3)_2$ (12 mg, 0.017 mmol, 20 mol%), CuI (7 mg, 0.035 mmol, 40 mol%) were introduced and put under an Argon atmosphere. A mixture of NEt_3 (5 mL) / THF (10 mL) previously distilled and degased was then added. The mixture was stirred at 70°C under argon during 18 h. After solvent evaporation the purple crude product was finally purified by column chromatography (SiO_2: from cyclohexane/dichloromethane (50/50) to dichloromethane/methanol (92/8)) yielding tweezers **1** as a purple solid (52 mg, 33%).

^{1}H NMR (400 MHz, CD_2Cl_2) δ 9.33 (dd, J = 0.7, 1.5 Hz, 2H, H_5), 8.79 (dd, J = 0.7, 5.0 Hz, 2H, H_3), 8.61 (s, 2H, H_2), 8.57 (s, 2H, H_9), 8.27 (d, J = 1.2 Hz, 2H, H_6), 8.25 (s, 2H, $H_{9'}$), 7.70 (d, J = 2.2 Hz, 2H, $H_{11'}$), 7.48 (m, 4H, H_{11-4}), 7.28 (d, J = 8.6 Hz, 2H, H_8), 7.12 (d, J = 2.5 Hz, 2H, H_{10}), 7.05 (d, J = 2.2 Hz, 2H, $H_{10'}$), 6.82 (dd, J = 1.2, 8.6 Hz, 2H, H_7), 1.61 (s, 18H, $H_{13'}$), 1.53 (s, 9H, H_1), 1.46 (s, 18H, $H_{12'}$), 1.33 (s, 18H, H_{12}), 1.13 (s, 18H, H_{13})

^{13}C NMR (100 MHz, CD_2Cl_2) δ 165.29, 164.53, 163.66, 157.10, 154.80, 150.21, 149.64, 147.74, 146.36, 145.30, 141.83, 141.70, 137.55, 137.44, 132.51, 131.66, 131.51, 129.59, 129.47, 128.91, 125.76, 124.40, 121.37, 120.99, 119.72, 118.42, 114.83, 94.30, 89.83, 36.81, 36.34, 36.06, 34.58, 34.35, 31.68, 31.53, 31.14, 30.62, 29.95.

ESI-HRMS *m/z* (%): $[M+Na]^+$ calc ($C_{95}H_{107}N_7Pt_2O_4$): 1823.7591 (100), found: 1823.7589 (100)

Titration procedures

^{1}H NMR titrations were performed using $CDCl_3$ dried over molecular sieves (4 Å) and passed through dried neutral aluminum oxide. Metal salts and tris(2-aminoethyl)amine were used without any purification. All solutions of tweezers, metal salts and ligands, used for titrations were prepared in volumetric flasks, and additions were made with Hamilton syringes.

Tweezers **1** opening: To 0.5 mL of closed tweezers **1** (1.0×10^{-3} M) dissolved in DMSO-d_6 in an NMR tube (5 mm), were added 0.2 eq of $ZnCl_2$ (4 µL of a 2.5×10^{-2} M solution in D_3CCN). After each metal addition, the tube was heated at reflux during 5 seconds, then cooled at room temperature, and the ^{1}H NMR spectrum was recorded.

Guest binding: To 0.5 mL of closed tweezers **1** (2.0×10^{-3} M) dissolved in $CDCl_3$ in an NMR tube (5 mm), were added coronene as a solid. After each metal addition, the tube was heated at reflux during 5 seconds, then cooled at room temperature, and the ^{1}H NMR spectrum was recorded.

UV-visible absorption spectra were recorded on a JASCO V-670 spectrophotometer at 25°C. $CHCl_3$ was dried over molecular sieves 4 Å and neutralized on neutral Al_2O_3. Metal salts were used without any purification. Solutions of tweezers, metals, used for titrations were prepared in volumetric flasks, and additions were made with Hamilton syringes. The metal salt concentrations in the stock solutions were checked by titration with a terpyridine solution. Curve fitting were performed by a nonlinear least-squares fit of the absorbance versus the concentration of guest added using the Matlab program developed by P. Thordarson.[4]
The titrations monitored by UV-Visible spectroscopy have been performed according to the following general procedure:

Tweezers **1** opening: To 3.0 mL of open tweezers (5.0×10^{-6} M) dissolved in $CHCl_3$ in a quartz cell (10 mm), were added 0.1 eq $ZnCl_2$ (3 µL of 1.0×10^{-3} M solution in H_3CCN). After each addition, a UV-Visible absorption spectrum (250 – 700 nm, 400 nm/min, 25°C) was recorded.

[Zn(**1**)]Cl_2 closing: To 3.0 mL of closed tweezers (5.0×10^{-6} M) dissolved in $CHCl_3$ in a quartz cell (10 mm), were added 0.1 eq tren (3 µL of 1.0×10^{-3} M solution in H_3CCN). After each addition, a UV-Visible absorption spectrum (250 – 700 nm, 400 nm/min, 25°C) was recorded.

Computational details

Calculations were performed with the Gaussian 09 software.[5] Complete geometry optimizations were carried out using the density functional theory method with the conventional Becke-3-Lee-Yang-Parr (B3LYP) exchange-correlation functional and 6-31G**/LanL2DZ. The platinum atoms were modeled using the effective core potential and the corresponding valence orbitals LanL2DZ in order to decrease the number of basis functions. The other atoms were described by the double zeta 6-31G** base which takes into account the polarization orbitals of all atoms, including hydrogen atoms. Vibrational analysis was performed at the same level in order to check the obtaining of a minimum on the potential energy surface.

Switching studies

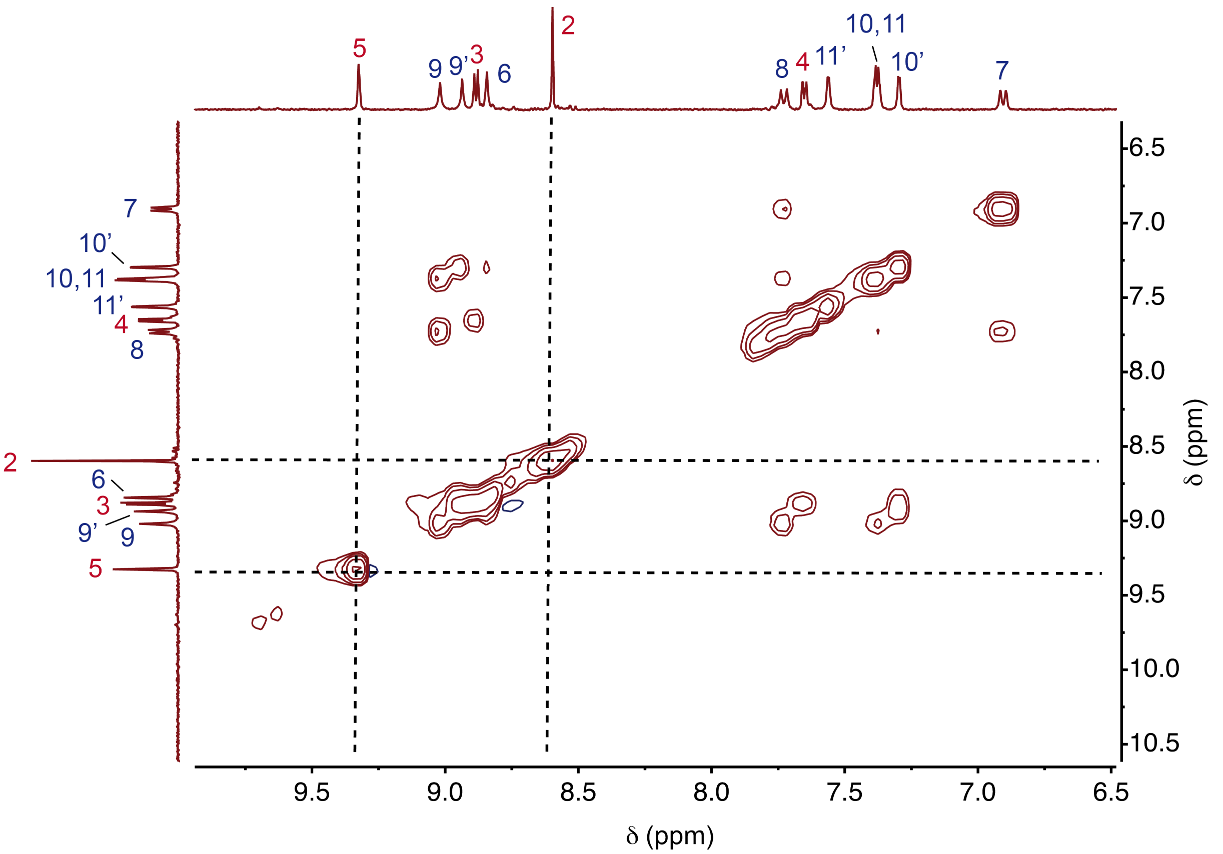

Figure S1: NOESY (400 MHz, 300 K) spectrum of Tweezers **1** in DMSO-d_6.

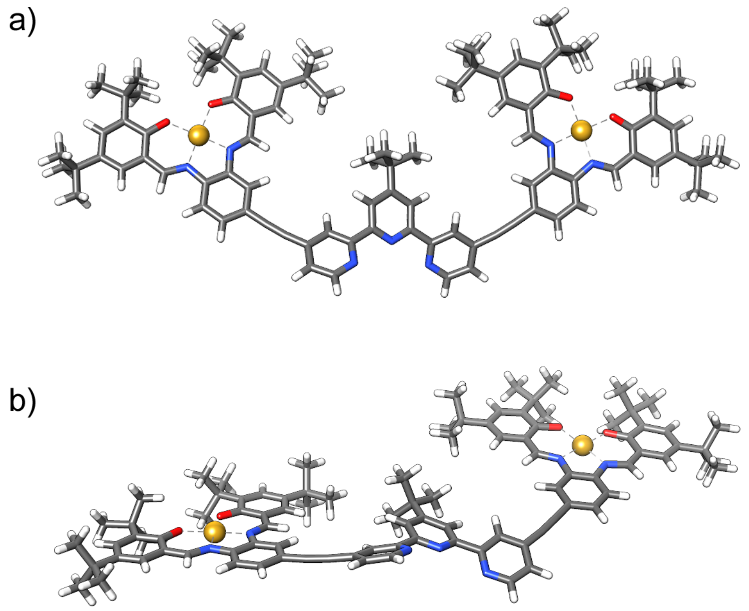

Figure S2: DFT optimized structure of W shaped conformation of **1** (B3LYP/ 6-31G**/LanL2DZ); a) top view and b) side view.

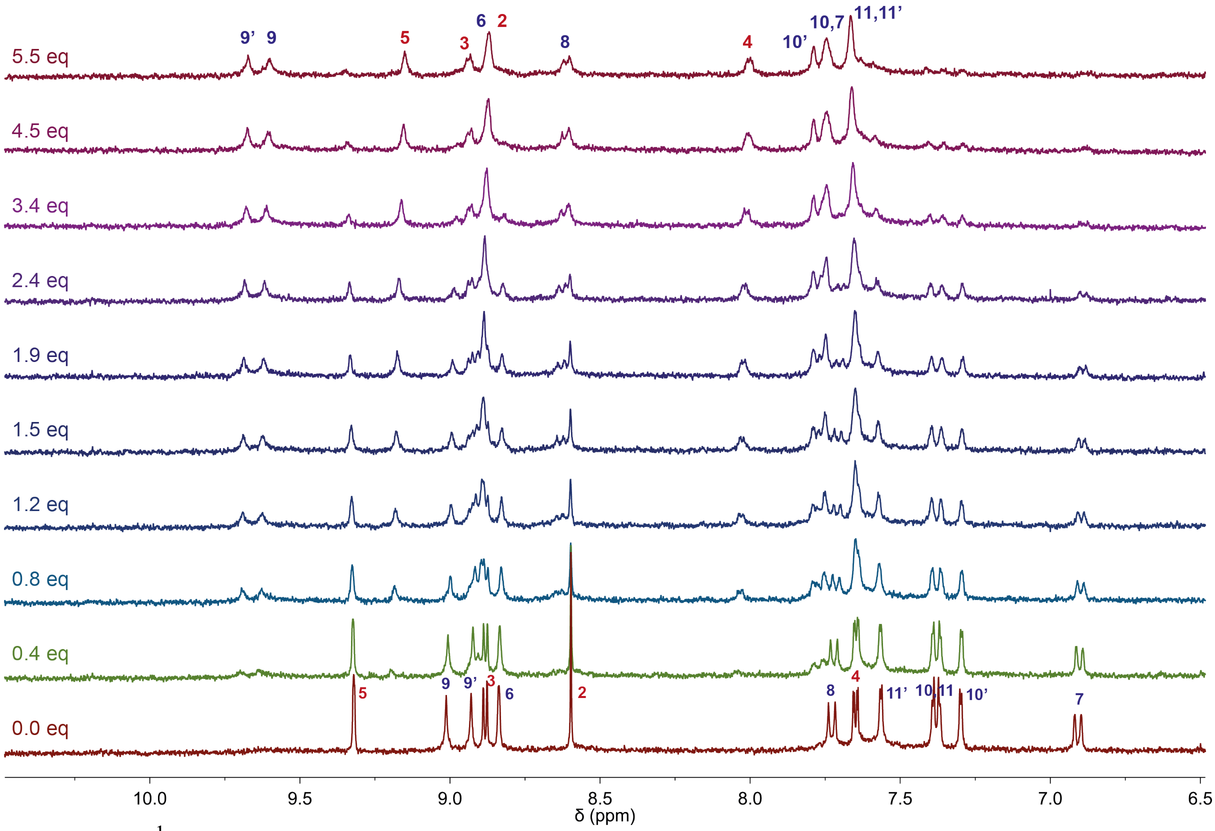

Figure S3: ^{1}H-NMR (400 MHz) titration of the opening of **1** by $ZnCl_2$ in DMSO-d_6 at 300 K.

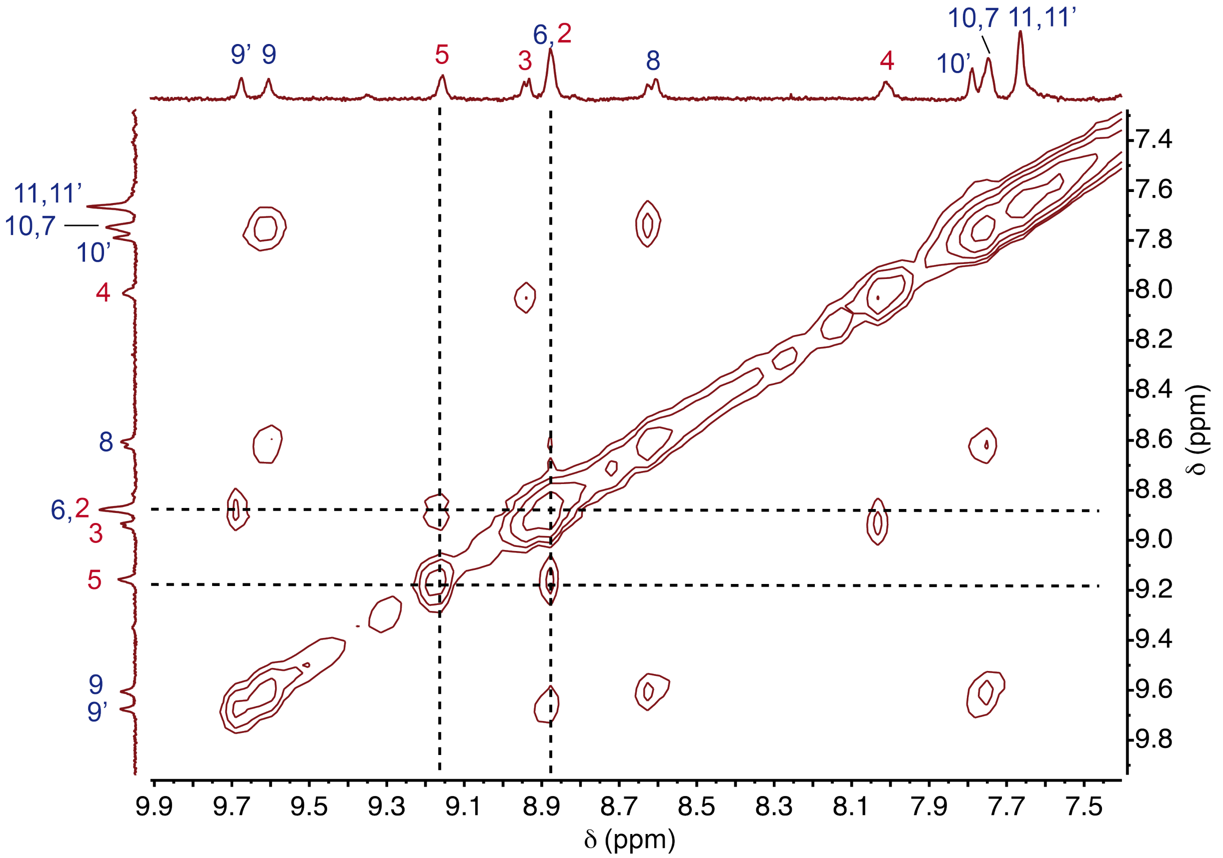

Figure S4: NOESY (400 MHz, 300 K) spectrum of $[Zn(\mathbf{1})_2]Cl_2$ in DMSO-d_6.

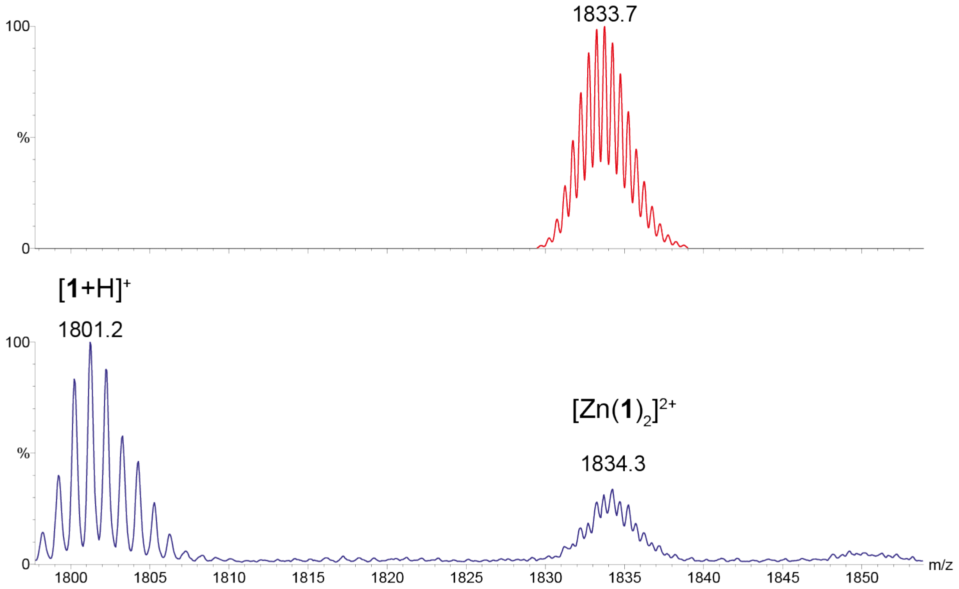

Figure S5: Mass spectrum $[Zn(\mathbf{1})_2]^{2+}$ experimental (bottom) and calculated (top).

Guest-binding studies

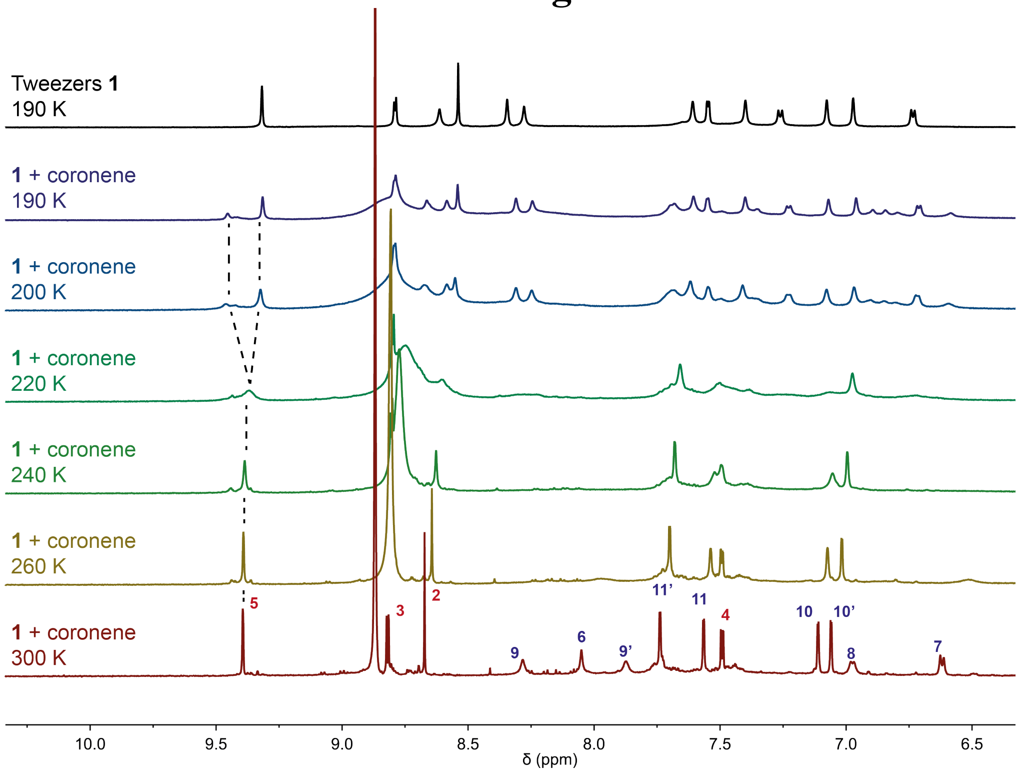

Figure S6: VT ^{1}H-NMR (600 MHz) of a 1:1.5 mixture of **1** and coronene in CD_2Cl_2.

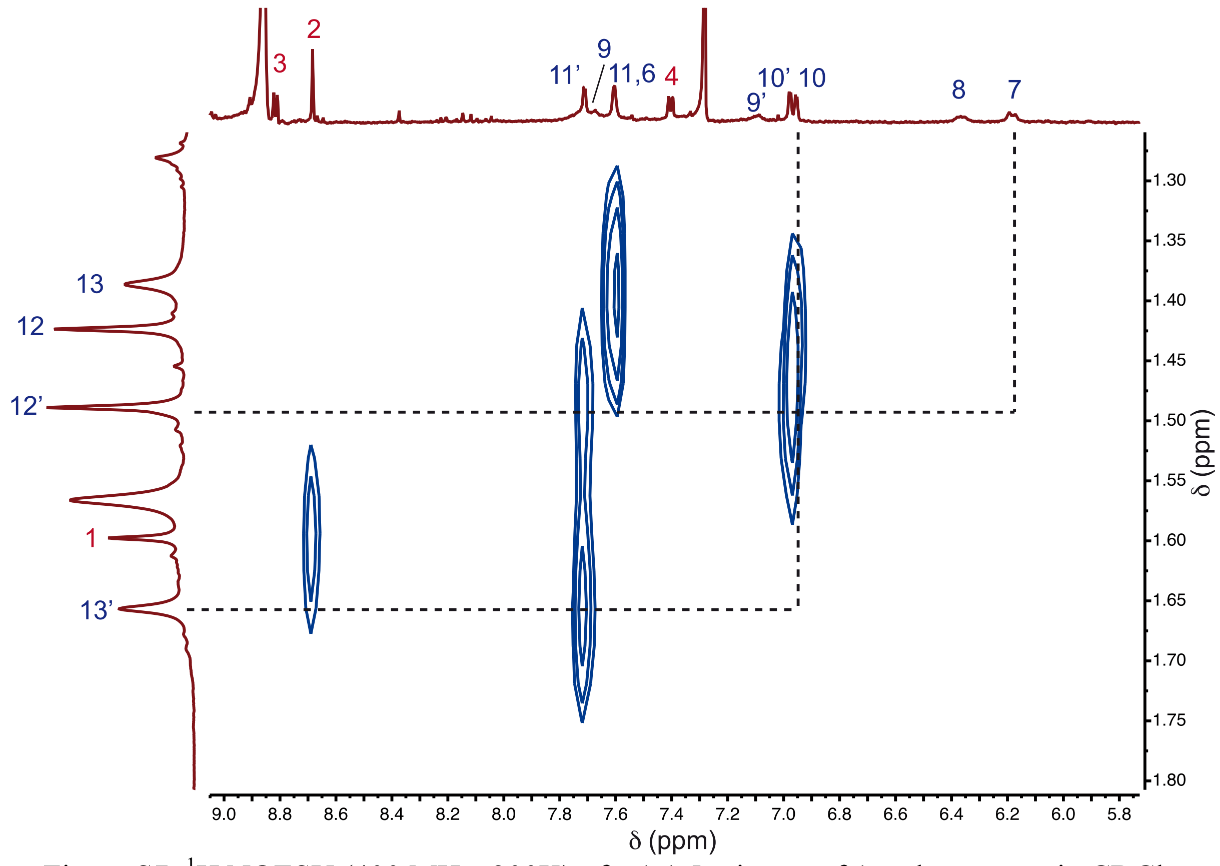

Figure S7: ^{1}H NOESY (400 MHz, 300K) of a 1:1.5 mixture of **1** and coronene in $CDCl_3$.

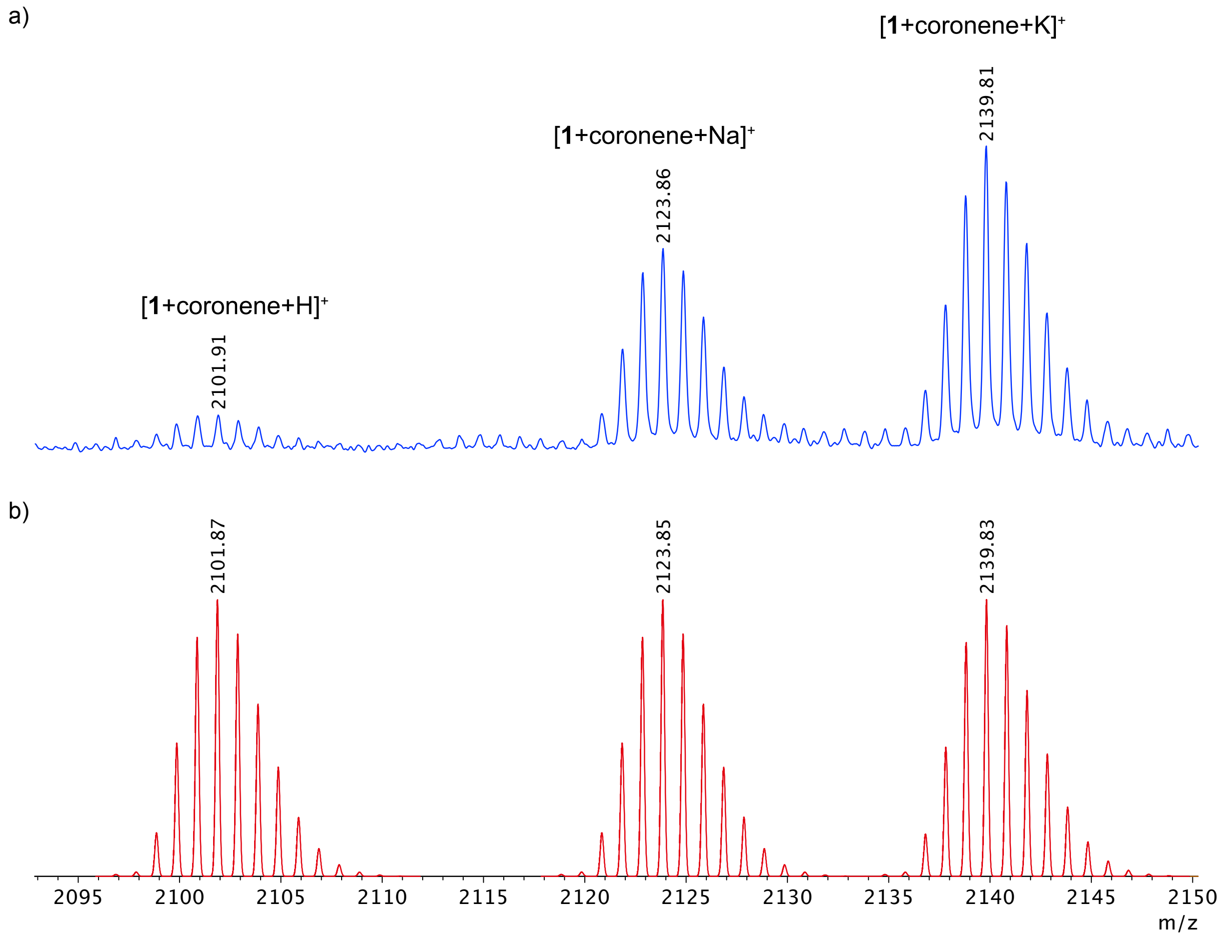

Figure S8: Mass spectrum of [coronene ⊂ **1**] ; a) experimental and b) calculated.

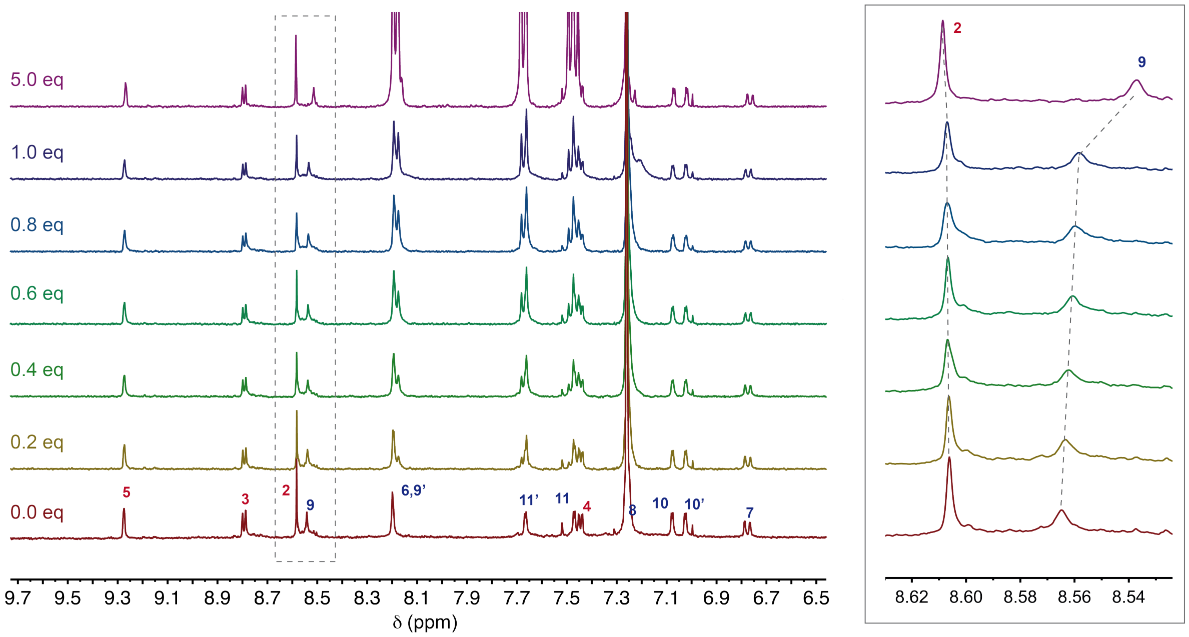

Figure S9: ^{1}H-NMR (400 MHz) titration of Tweezers **1** (2.0 mM) with perylene in $CDCl_3$ at 300 K.

References

[1] B. Doistau, A. Tron, S. A. Denisov, G. Jonusauskas, N. D. McClenaghan, G. Gontard, V. Marvaud, B. Hasenknopf, G. Vives, *Chem. Eur. J.* **2014**, *20*, 15799-15807.

[2] H. Ozawa, T. Kuroda, S. Harada, H. Arakawa, *Eur. J. Inorg. Chem.* **2014**, *2014*, 4734-4739.

[3] E. C. Constable, N. Hostettler, C. E. Housecroft, P. Kopecky, M. Neuburger, J. A. Zampese, *Dalton Trans.* **2012**, *41*, 2890-2897.

[4] P. Thordarson, *Chem. Soc. Rev.* **2011**, *40*, 1305-1323.

[5] M. J. Frisch, G. W. Trucks, H. B. Schlegel, G. E. Scuseria, M. A. Robb, J. R. Cheeseman, G. Scalmani, V. Barone, B. Mennucci, G. A. Petersson, H. Nakatsuji, M. Caricato, X. Li, H. P. Hratchian, A. F. Izmaylov, J. Bloino, G. Zheng, J. L. Sonnenberg, M. Hada, M. Ehara, K. Toyota, R. Fukuda, J. Hasegawa, M. Ishida, T. Nakajima, Y. Honda, O. Kitao, H. Nakai, T. Vreven, J. A. Montgomery Jr., J. E. Peralta, F. Ogliaro, M. J. Bearpark, J. Heyd, E. N. Brothers, K. N. Kudin, V. N. Staroverov, R. Kobayashi, J. Normand, K. Raghavachari, A. P. Rendell, J. C. Burant, S. S. Iyengar, J. Tomasi, M. Cossi, N. Rega, N. J. Millam, M. Klene, J. E. Knox, J. B. Cross, V. Bakken, C. Adamo, J. Jaramillo, R. Gomperts, R. E. Stratmann, O. Yazyev, A. J. Austin, R. Cammi, C. Pomelli, J. W. Ochterski, R. L. Martin, K. Morokuma, V. G. Zakrzewski, G. A. Voth, P. Salvador, J. J. Dannenberg, S. Dapprich, A. D. Daniels, Ö. Farkas, J. B. Foresman, J. V. Ortiz, J. Cioslowski, D. J. Fox, Gaussian, Inc., Wallingford, CT, USA, **2009**.

NMR spectra of compounds

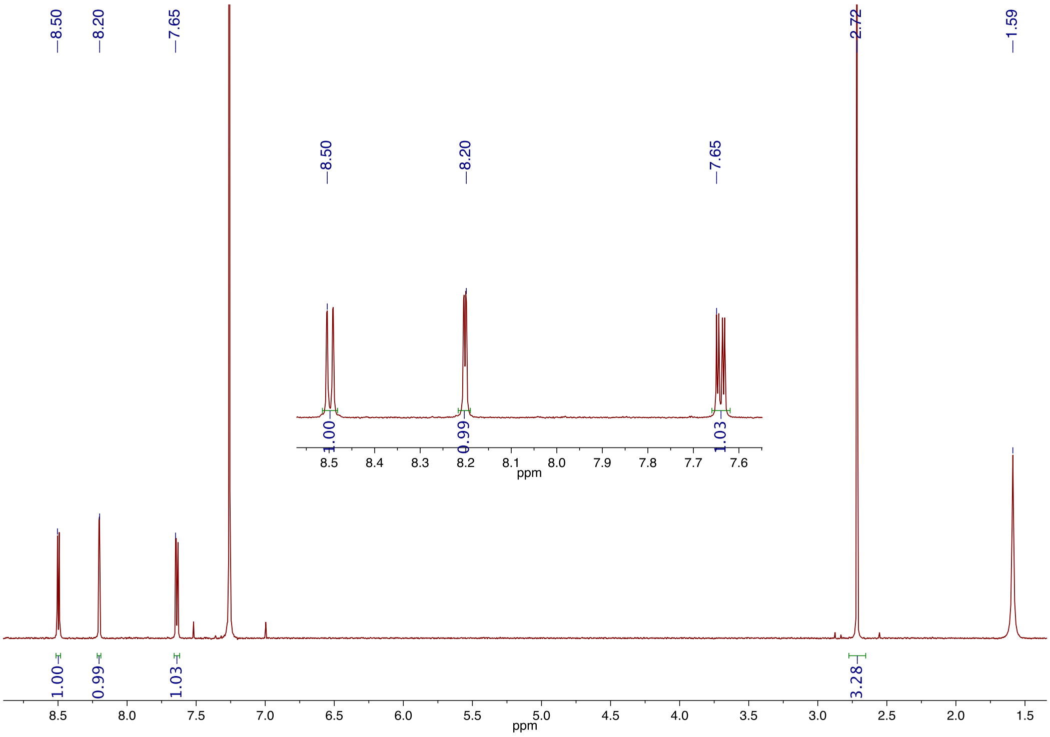

^{1}H NMR spectrum of *4-bromo-2-acetylpyridine* **3** (300 K, 400 MHz, $CDCl_3$)

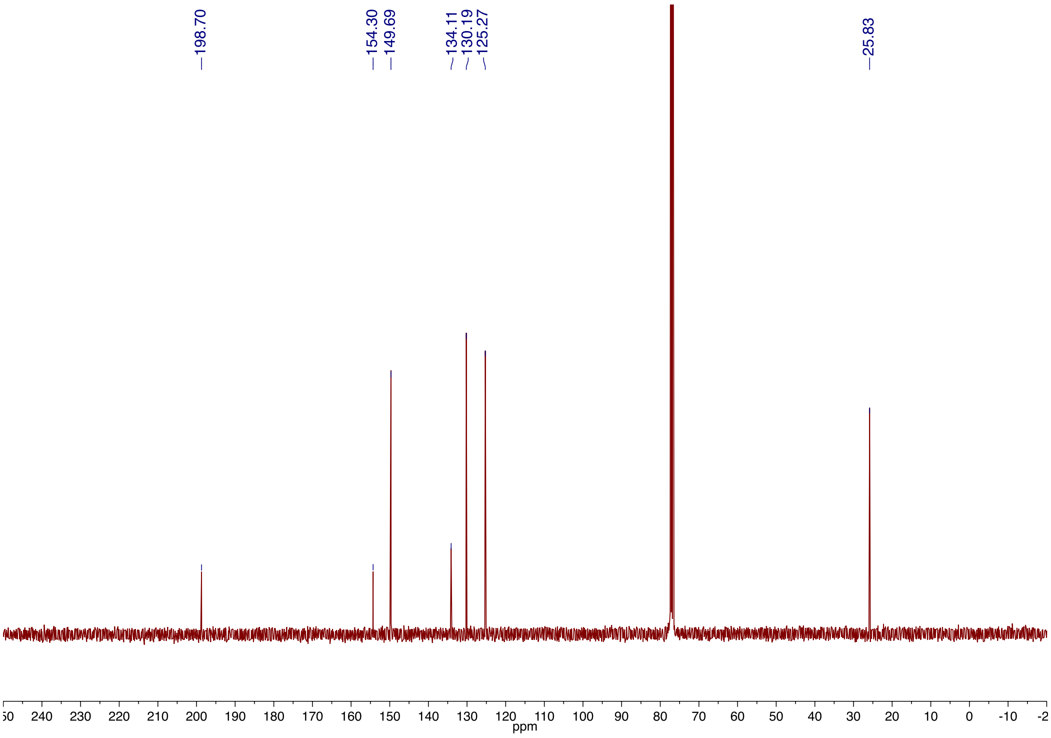

^{13}C NMR spectrum of *4-bromo-2-acetylpyridine* **3** (300 K, 100 MHz, $CDCl_3$)

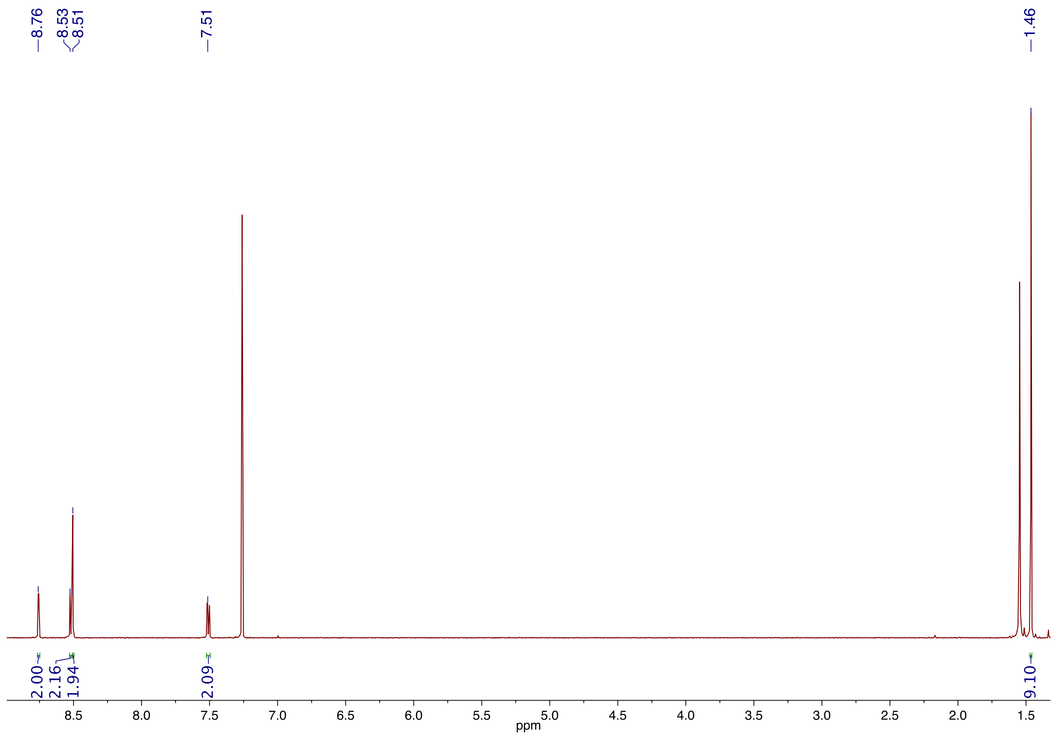

^{1}H NMR spectrum of *4,4''-Dibromo-4'-(tert-butyl)-2,2':6',2''-terpyridine **4*** (300 K, 400 MHz, $CDCl_3$)

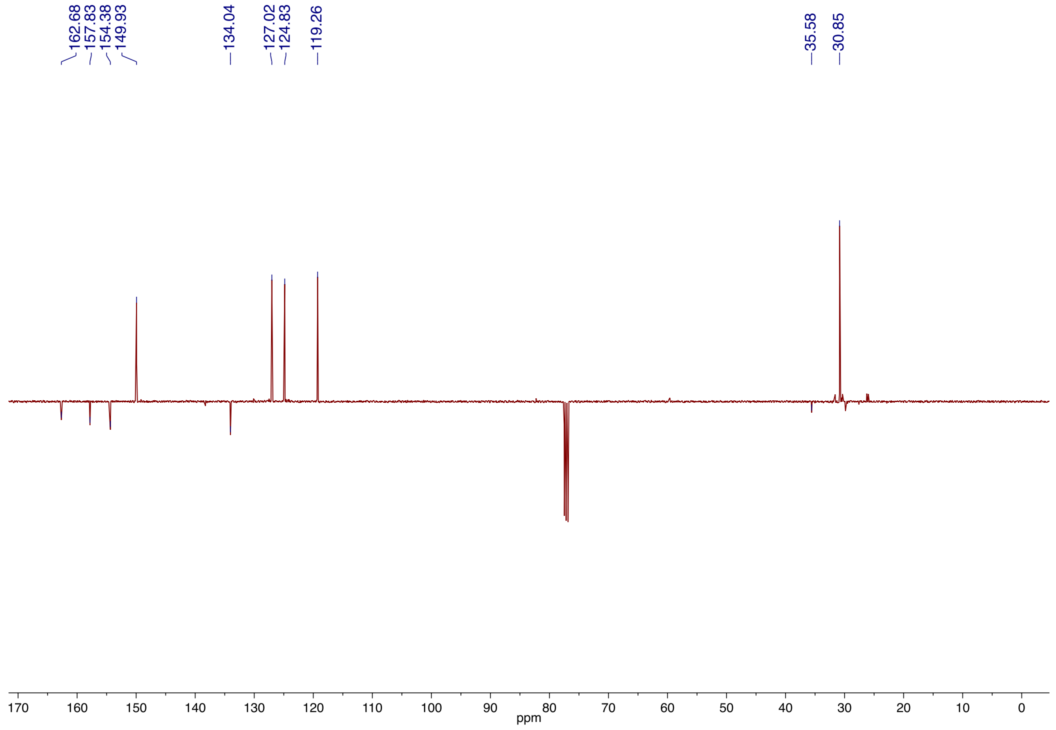

^{13}C JMOD NMR spectrum of *4,4''-Dibromo-4'-(tert-butyl)-2,2':6',2''-terpyridine **4*** (300 K, 100 MHz, $CDCl_3$)

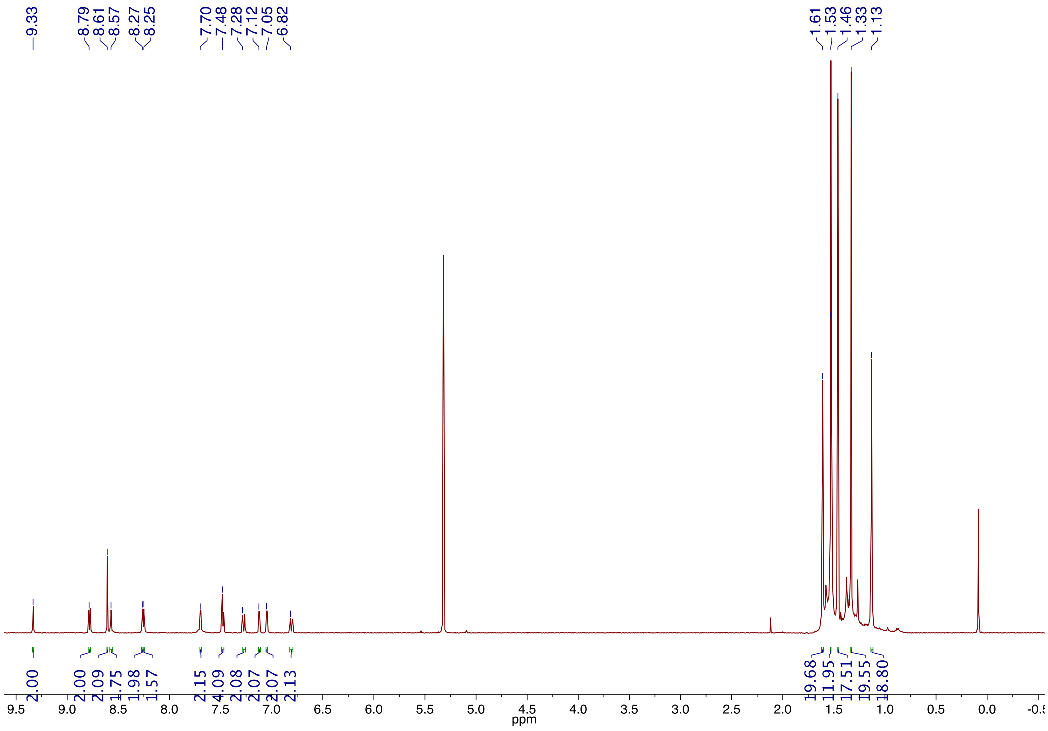

^{1}H NMR spectrum of *tweezers **1*** (300 K, 400 MHz, CD_2Cl_2)

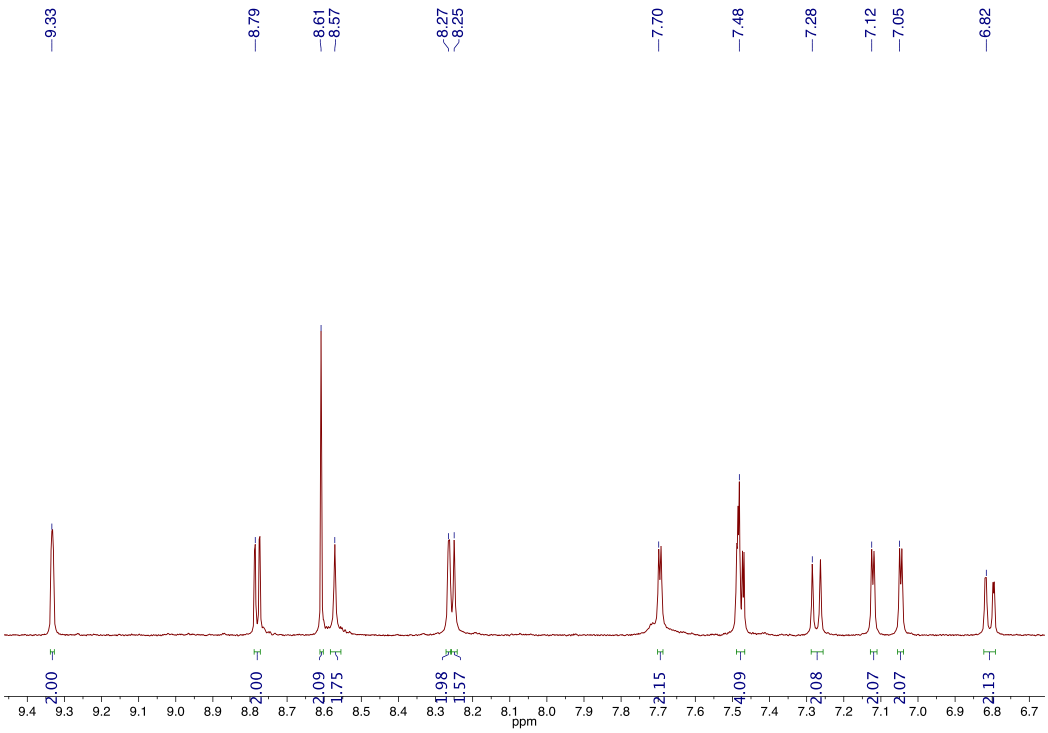

^{1}H NMR spectrum of *tweezers **1*** (300 K, 400 MHz, CD_2Cl_2)

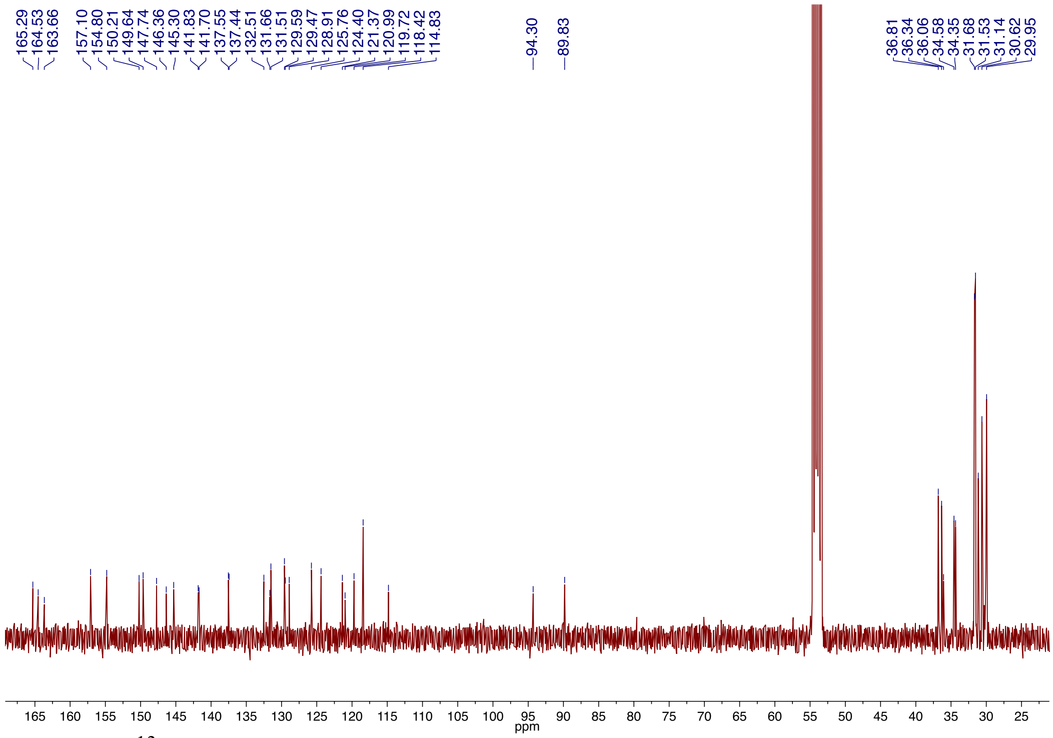

^{13}C NMR spectrum of *tweezers **1*** (300 K, 100 MHz, CD_2Cl_2)
